# Cyclosporine A Decreases Dryness-Induced Hyperexcitability of Corneal Cold-Sensitive Nerve Terminals

**DOI:** 10.3390/ijms241613025

**Published:** 2023-08-21

**Authors:** Andrea Gyenes, Zsófia Tapasztó, Susana Quirce, Carolina Luna, Laura Frutos-Rincón, Juana Gallar, M. Carmen Acosta, Illés Kovács

**Affiliations:** 1 Department of Ophthalmology, Faculty of Medicine, Semmelweis University, 1085 Budapest, Hungary; 2 Instituto de Neurociencias, Universidad Miguel Hernández—CSIC, 03550 San Juan de Alicante, Spaincarolina.luna@umh.es (C.L.); l.frutos@umh.es (L.F.-R.); mcarmen.acosta@umh.es (M.C.A.); 3 Instituto de Investigación Sanitaria y Biomédica de Alicante-ISABIAL, 03010 Alicante, Spain; 4 Department of Ophthalmology, Weill Cornell Medical College, New York, NY 10021, USA; 5 Department of Clinical Ophthalmology, Faculty of Health Sciences, Semmelweis University, 1088 Budapest, Hungary

**Keywords:** dry eye, cold thermoreceptors, cyclosporine A, corneal nerves

## Abstract

Cyclosporine A (CsA) is used for the treatment of dry eye (DE) with good clinical results, improving tear secretion and decreasing subjective symptoms. These effects are attributed to the improved tear film dynamics, but there are no data on the effect of CsA on the abnormal sensory nerve activity characteristic in DE. Our purpose was to evaluate the CsA effect on the enhanced activity of corneal cold thermoreceptors in a tear-deficient DE animal model using in vitro extracellular recording of cold thermoreceptors nerve terminal impulses (NTIs) before and in the presence of CsA. NTI shape was also analyzed. Blinking frequency and tearing rate were also measured in awake animals before and after topical CsA. CsA increased the tearing and blinking of treated animals. CsA significantly decreased the peak response to cold of cold thermoreceptors. Neither their spontaneous NTIs discharge rate nor their cooling threshold were modified. CsA also seemed to reverse some of the changes in NTI shape induced by tear deficiency. These data suggest that, at least in part, the beneficial clinical effects of CsA in DE can be attributed to a direct effect on sensory nerve endings, although the precise mechanisms underlying this effect need further studies to be fully clarified.

## 1. Introduction

The Dry Eye Workshop (TFOS DEWS II) defines dry eye disease (DED) of the ocular surface to be a result of several factors, the etiology of which is typically caused by a loss of homeostasis of the tear film, leading to ocular symptoms caused by the resulting hyperosmolarity and instability of the tear film, inflammation and damage of the ocular surface, and abnormalities in the neurosensitivity [1]. A fundamental complaint reported in dry eye disease patients is ocular discomfort [1], presumably due to the activation of nociceptive sensory nerves at the ocular surface [2].

Ocular sensory neurons are classified as polymodal nociceptor neurons, cold thermoreceptor neurons, and selective mechano-nociceptor neurons [3,4,5]. There is significant evidence suggesting that cold thermoreceptors have the ability to detect small changes in both temperature and osmolarity; this makes it likely that their primary role is providing information about the dryness level of the eye surface [6], regulating tear secretion to maintain the adequate moistness of the ocular surface [7] and contributing to the reflex control of blinking and basal tear production [8]. The characteristically unpleasant dryness sensation reported by patients with DED can be explained by the hyperexcitability of cold thermoreceptors [9].

To reduce symptoms of irritation, patients with dry eyes generally use artificial tears; however, a majority of patients report aberrant ocular surface sensations despite the frequent use of tear supplements [10,11]. For patients with DED refractory to conservative treatments, such as ocular lubricants and lid hygiene, the use of topical cyclosporine A (CsA) is recommended [12,13]. Both animal [14,15,16,17,18] and human [18,19,20] studies support the benefit of using CsA for its effect on the underlying inflammatory condition of DED. A large-scale multicenter, vehicle-controlled, and dose-ranging (phase 2) preliminary investigation of the clinical trial demonstrated the safety and efficacy of CsA treatment in moderate to severe DED in 2000 [19], leading to its FDA approval in October 2003 [21]. As an immunomodulating agent, CsA has numerous molecular influences on DED immunopathophysiology. A meta-analysis comparing topical CsA 0.05% to control showed improvement on several parameters such as Ocular Surface Disease Index (OSDI) scores, tear breakup time, Schirmer I score, corneal fluorescein staining, and goblet cell density [22].

CsA has been reported as an immunomodulator affecting reinnervation after transection of corneal stromal nerves [23]. The inhibiting actions could be attributed, at least partially, to the reduced expression of proinflammatory, neurogenic cytokines such as IL-6 [24]. CsA also shows concentration-dependent neurotoxicity, and its systemic administration has been described to have neurological side effects [25].

The beneficial effects of CsA treatment are primarily attributed to the improved tear film dynamics, and there are no data on the possible effect of CsA on the altered sensory nerve activity, a characteristic feature of chronic DED. The purpose of this study was to evaluate the effect of CsA on the enhanced activity of corneal cold nerve terminals in an animal model of tear-deficient dry eye.

## 2. Results

### 2.1. Effects of CsA on Cold Thermoreceptors’ Nerve Activity

We first recorded in intact eyes the spontaneous activity at the basal temperature of 34 °C of the corneal nerve terminals of cold thermoreceptor neurons and their marked frequency increase (peak response to cold) caused by cooling ramps from 34 °C to 15 °C. We then confirmed that, as in previous works, cold nerve terminals from tear-deficient animals present an increased spontaneous firing frequency at 34 °C, as well as comparatively larger responses to cooling ramps compared with cold nerve terminals recorded from control, intact corneas. In intact corneas, perfusion with a solution containing CsA at 50 µM had no significant effects in any of the parameters measured to characterize the spontaneous and the stimulus-evoked activity, although a small decrease in the peak response to cold was observed (Table 1). After washing the drug for 15 min, the values obtained were similar to those obtained before CsA (Spontaneous activity: 6.8 ± 0.8 imp/s; Cooling threshold: 31.9 ± 0.5 °C; Peak response: 26.2 ± 5.4 imp/s).

**Figure 1:**
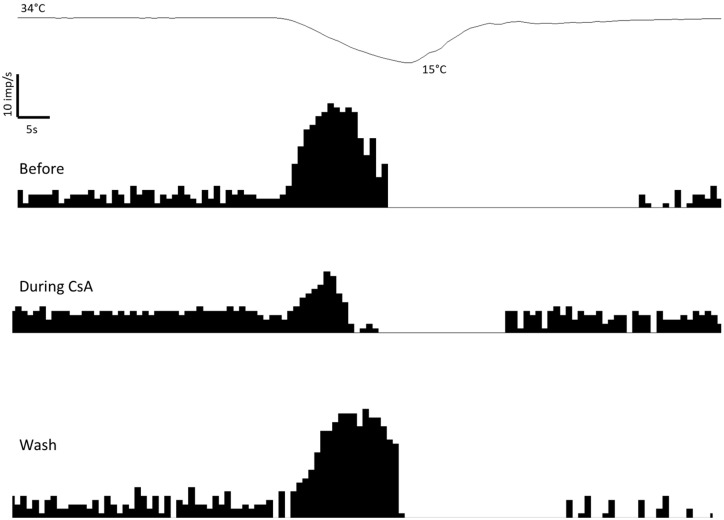
Effects of cyclosporine A (CsA) on the spontaneous and cold-evoked activity of a single cold thermoreceptor nerve terminal recorded from a tear-deficient cornea. The discharge rate at 34 °C and during the cooling ramp from 34 °C to 15 °C performed before, during perfusion with solution containing CsA at 50 µM, and 15 min after washing with control solution is shown. Upper trace: temperature of the bath solution. Lower traces: frequency histogram of the impulse discharge in imp/s.

**Figure 2:**
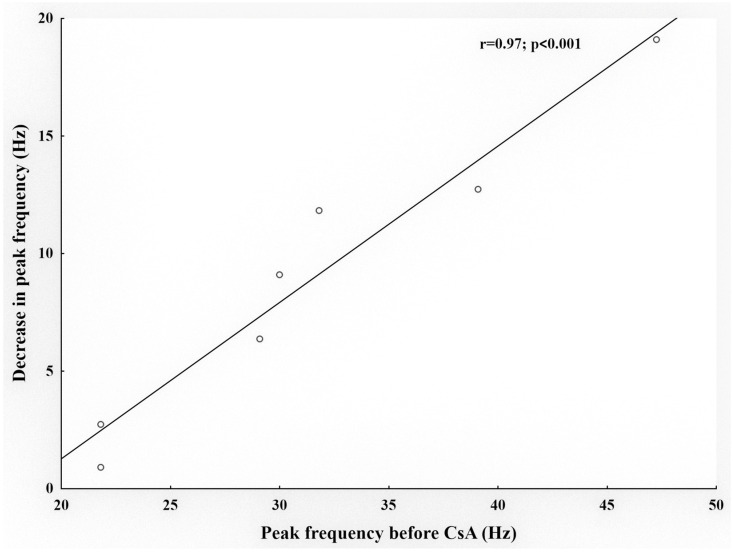
Correlation between the peak frequency in response to cold before CsA application and the decrease in the peak response to cold during the perfusion with CsA in tear-deficient corneas. Notice that in nerve terminals with a high peak frequency in response to cooling, the effect of CsA was greater than in terminals with low peak frequencies, the correlation between both parameters being statistically significant (*p* < 0.001).

In tear-deficient corneas, the perfusion with 50 µM CsA resulted in a significant decrease in the peak response to the cold of cold nerve terminals (Table 1). This effect was reversed in the majority of the units explored 15–30 min after washing (Figure 1). The spontaneous activity and cooling threshold were not affected by CsA (Table 1; Figure 1). The decrease in peak response to cooling during the perfusion with 50 µM CsA in tear-deficient corneas was strongly correlated with the peak response to cooling before CsA application (Figure 2). This means that the greater the response to cold in tear-deficient terminals, the greater the effect of CsA decreasing it.

### 2.2. Effects of CsA on the Shape of NTIs of Cold Thermoreceptors

In control corneas, the parameters measured to characterize the shape of NTIs fired spontaneously (at a basal temperature of 34 °C) were not modified during the perfusion with CsA (Table 2).

The changes in NTI shape of cold thermoreceptors in tear-deficient animals have not been previously studied. We found that NTI shape from tear-deficient corneas presented already a significant decrease in the duration of the NTI compared to the control (Table 2), which could be related to the increased spontaneous activity present in tear-deficient cold thermoreceptors (Table 1). Regarding the effect of the perfusion with 50 µM CsA in tear-deficient corneas, there were no significant changes in any of the measured parameters (Table 2).

### 2.3. Effect of CsA on Blinking and Tearing

In control animals, CsA increased significantly in blinking and tearing (Table 3). In four eyes blinking and tearing measurements were repeated 10 min after the CsA application, and basal values were recovered (0.3 ± 0.3 blinks/min; 12.0 ± 3.2 mm; *n* = 4). In tear-deficient animals, a slight increase in blinking was observed (Table 3) above the increased basal blinking in DE animals. Although tearing was dramatically reduced in tear-deficient animals after the excision of the main lacrimal gland, a slight increase in tear rate was also observed during CsA treatment (Table 3). Ten min after the CsA instillation, basal values of blinking and tearing were recovered in tear-deficient animals (7.7 ± 2.6 blinks/min; 5.2 ± 0.8 mm; *n* = 6).

## 3. Discussion

In this study, we confirmed our previous results that corneal cold nerve terminals have increased spontaneous activity and are more sensitive to cold stimulation four weeks after lacrimal gland removal in guinea pigs. The main outcome of this work is that, for the first time, we described here that CsA has an acute effect on corneal nerves and significantly reduces the increased neural activity of corneal cold receptors in tear-deficient animals. However, the precise mechanisms through which CsA has this acute effect on corneal nerves still need to be further studied.

Ocular discomfort is reported to be a major complaint of DED [9,26,27]. The activation of corneal sensory nerves (polymodal and mechano- nociceptors and cold thermoreceptors) is responsible for the sensations evoked from the ocular surface [4,5]. Both polymodal and mechano-nociceptors are sensitized during DED [9], being responsible, in part, for the discomfort and pain reported.

Of all the ocular surface sensory neurons, cold thermoreceptors represent 10–15% of them. At temperatures that are stable, close to that of the ocular surface (34–35 °C), most corneal cold thermoreceptor fibers repeatedly generate action potentials; their activity is increased and decreased by moderate cooling and heating, respectively [28,29]. Dysfunction of corneal cold thermoreception likely contributes to the etiology of DED [9,30,31,32]. This theory proposes that the strong activation of cold thermoreceptors of the dry eye surface elicits a sense of irritative dryness sensation [8].

Animal studies performing lacrimal gland removal showed the effects of chronic deficiency in tear production on corneal sensory receptors. In both rats and guinea pigs, decreased tear production sensitizes cold thermoreceptors, with both a shift in the cooling threshold to warmer values and an increased peak frequency of nerve activity evoked during cooling [9,33]. In guinea pigs, there is a gradual increase in the level of spontaneous nerve activity of cold thermoreceptors, and the sensitization of cold thermoreceptors induced by tear deficiency is associated with morphological changes to the corneal innervation, suggestive of nerve damage [9]. No changes were found in the cold-induced inward current during the electrophysiological investigation of corneal cold-sensitive neurons isolated from guinea pigs with removed lacrimal glands [9]. This finding suggests that the increased activity of cold nerve terminals is not due to increased expression of TRPM8 channels; instead, there are increases in Na^+^ channel currents and decreases in K^+^ channel currents in corneal cold thermosensitive neurons, the changes that would increase neuron excitability by reducing the voltage threshold for action potential initiation [34].

Topical therapies that are available for DED include immunosuppressive agents (corticosteroids), immunomodulatory agents (0.05% cyclosporine ophthalmic emulsion and 0.09% cyclosporine ophthalmic solution), and the recently available LFA-1 antagonist lifitegrast ophthalmic solution at 5% [13]. According to previous reports, a cardinal effect of CsA is inhibiting T cell activation, decreasing cyclophilin-mediated gene transcription of IL-2 and IL-6 [35]. The action of CsA involves the regulation of T-cell activation through calcineurin, a major regulator of IL6 expression [36]. T cells are not the sole target of cyclosporin; it has an impact on adaptive immune responses, dendritic cells, macrophages, neutrophils, and mitochondrial DAMPs [37].

Besides the inflammatory role, the expression of IL-6 plays a promoting role in nerve regeneration [38]. Nerve regeneration is positively influenced by the inflammatory process after superficial corneal injury; it is to be noted that CsA only has an effect on immune cells, predominantly T cells, and does not have a significant effect on the function of resident corneal cells nor on corneal wound healing [39]. In addition to its immunomodulatory actions, CsA has a direct inhibiting effect on axonal growth; CsA downregulates the expression of neurotrophins, such as BDNF and NT5 [40], decreases the expression of NFATC1 and MAPK14, and significantly increases the expression of FOS and GAP43 [23]. CsA induces nerve growth factor (NGF) expression via activation of MAPK p38-NFAT5 axis on the human epithelial cell line [41].

The neuroprotective effect of cyclosporin was confirmed if given in as early as 30 min after the injury by systemic administration [42]. The probable axonal regenerative effect could be explained by the inhibition of white blood cell function and inhibiting calcium-dependent cell injury. Low-dose subcutaneous administration of CsA increased fiber density [43]. The neuroprotective action of local CsA application in peripheral nerve injuries was also demonstrated [44]. Regarding the functional and biochemical effect of CsA, the nerve regeneration process was accelerated by local administration of CsA after nerve transection [45].

Voltage-gated sodium channels are major contributors to action potential electrogenesis, and their modulation can regulate the function of excitable cells [46]. Mechanisms that regulate the current density and biophysical properties of Na_V_1.8 are a major focus due to investigations of this channel’s role in inflammatory and neuropathic pain. Calmodulin binding to Na_V_1.8 in native neurons can regulate the current density and the ability of this channel to respond to high-frequency depolarizations [46].

Calcineurin inhibition by CsA has been described to affect the regulation of neurotransmitter release in sensory nerve endings. A calcineurin-synapsin negative feedback mechanism normally prevents excessive calcium-dependent depolarization of afferent endings. CsA, through synapsin phosphorylation–dephosphorylation, enhances vesicle-mediated exocytosis that augments membrane depolarization [47]. Calcineurin inhibitor CsA inhibits voltage-gated calcium channel-dependent LTP (long-term potentiation) [48]. CsA showed an inhibitory effect similar to FK506 on potassium channel blocker tetraethyleneammonium (TEA)-induced LTP, suggesting that calcineurin is involved in the processes of this kind of synaptic plasticity.

Although it is used in clinical practice, the mechanism of action of CsA has not been fully elucidated [12]. The long-term application of CsA results in increased natural tear production and increased goblet-cell density [49].

In our experiments, we recorded the spontaneous and evoked activity of cold thermoreceptors, which are sensitized in DED. We found that CsA has no effect on cold thermoreceptors activity from controls corneas, although it decreased the enhanced response to cold in tear-deficient corneas. Therefore, the CsA is having an acute effect only on the abnormal activity of cold thermoreceptors, reversing their increased evoked activity. Interestingly, this reversing effect significantly correlated with their abnormal activity. Trying to clarify its mechanism of action, we analyzed the changes in the shape of NTI fired during spontaneous activity. Our technique allows for the extracellular recording of the membrane potential changes produced at the nerve terminal, which are proportional to the net membrane current. So, the shape of the NTI represents the first derivative of the membrane voltage change, which is outward during the spike depolarization (generating a positive peak) and inward during the spike repolarization (recorded as a negative peak) [50]. Using dedicated software Spike2 (see Section 4), we have studied several parameters to define the NTI shape.

This is the first time that the NTI shape has been analyzed in tear-deficient cold nerve terminals. The changes in cold thermoreceptors’ NTI shape during the spontaneous activity mostly reflect the activity of K^+^ [51] channels. This is because, although Na+ channels are present in nerve terminals, they make only a minor contribution to the shape, probably because they are inactivated [51]. As there are increases in Na^+^ channel currents and decreases in K+ channel currents in tear-deficient cold thermoreceptors [9], it was expected that NTI recorded in tear-deficient corneas had already significant changes in the NTI shape compared to control, reflecting the reduced K^+^ currents. However, the results should be taken cautiously because we only could analyze the activity of a reduced number of tear-deficient nerve terminals, the small changes we found fit with the available information. We found a decrease in the NTI amplitude, as has been observed when K^+^ currents are blocked with different drugs [28]. Moreover, the downregulation of K^+^ currents and/or K^+^ channel expression is a general phenomenon in peripheral nociceptive fibers as a mechanism of sensitization, as observed in our tear-deficient animals. We also observed a significant decrease in the NTI duration that cannot be attributable to a decrease in the K^+^ currents because an increase in the NTI duration is produced by K^+^ blockers [28]. Overall, our data on the NTI shape support that part of the changes in the activity of cold thermoreceptors under tear-deficiency conditions are attributable to a decrease in K^+^ currents, but we cannot discard the contribution of changes in other currents.

Regarding the effect of CsA on NTI shape, no significant changes were observed in control corneas, but in tear-deficient corneas it seems to be a small effect, reversing the decrease in NTI duration in DE, although the decrease in NTI amplitude is still present. We can only guess that the effect of CsA on tear-deficient terminals should not be through an effect on K^+^ currents. This is in line with previous reports on rabbit coronary arterial smooth muscle cells, where CsA did not affect the Kv currents [51]. On the other hand, a direct effect of CsA on TRPM8 should also be discarded, as in TRPM8-transfected HEK293 cells, CsA application did not affect cold-evoked calcium responses [52]. Nevertheless, new experiments using pharmacological tools should be conducted in order to deeper investigate the mechanisms.

According to our results and previous reports [53] acute effect of CsA increased blinking and tearing in both control and tear-deficient guinea pigs, although the mechanism of this effect is not clear. It is already known that the spontaneous activity of cold thermoreceptors is behind the control of basal tearing and blinking [54,55]. The present study rules out that CsA is increasing tearing and blinking through the activation of cold thermoreceptors, as CsA increases blinking and tearing in control corneas, where it has no effect on cold thermoreceptors’ activity. The possibility exists that CsA is acting directly on the lacrimal glands and/or also activating nociceptors from the cornea and/or conjunctiva, most probably polymodal nociceptors, which are known to activate reflex tearing and blinking [56,57]. Studies about the effect of CsA on ocular surface nociceptors should be carried out. CsA protects human conjunctival epithelial cells via its anti-apoptotic action and improves conjunctival goblet cell density and corneal surface integrity via its immunomodulatory activities [13], but none of these mechanisms would explain such an acute and quick effect observed both in vivo and ex vivo.

As a summary, here we described for the first time that in tear-deficient animals, CsA significantly reduced the augmented activity of corneal cold receptors. The clinical relevance of these findings is that the topical application of CsA might result in a normalization of the aberrant corneal cold thermoreceptor activity, which is the presumed source of abnormal ocular surface sensations experienced in dry eye disease, without analgesic effect on corneal cold nerves. Although deeper analysis should be performed to clarify the precise CsA mechanisms explaining the observed acute effects of CsA, clinical studies are recommended as this beneficial effect would be better described when the effect of different concentrations of topical CsA on subjective symptoms of the dry eye could be analyzed. Our future clinical studies aim to examine whether the addition of low-dose CsA to artificial tears can lead to a more pronounced decrease in dry eye symptoms due to the combined effect of improvement in tear film dynamics and direct pharmacological modulation of the corneal nerve hyperexcitability responsible for hyperalgesia and pain in dry eye disease.

## 4. Materials and Methods

### 4.1. Animals

Dunkin Hartley guinea pigs of both sexes weighing 200–300 g were used. Animals were kept in individual cages with free access to food and water and were maintained under day–night cycles. This study was carried out in accordance with the NIH Guide for the Care and Use of Laboratory Animals, the European Union Directive (2010/63/EU), and the Spanish regulations on the protection of animals used for research, and followed a protocol approved and supervised by the Committee of Ethics and Integrity in Research (CEIR) of the University Miguel Hernández.

### 4.2. Surgical Procedure

Removal of the main lacrimal gland was performed following the technique previously described in detail [10]. Animals were anesthetized with an i.p. injection of ketamine (90 mg/kg) and xylazine (5 mg/kg). After performing a unilateral 8 mm skin incision on the temporal side of the face, posterior to the lateral canthus, the fibrous capsule of the exorbital lacrimal gland was exposed and dissected. Then, the lacrimal gland was excised in toto in 18 animals. At the end of the operation, a drop of antibiotic solution (3 mg/mL tobramycin) was applied to the surgical area. The skin incision was then sutured using a 6.0 braided silk suture. Animals were housed individually, and conjunctival and corneal signs were checked regularly. A group of non-operated guinea pigs of both sexes of similar age and weight was used as a control group (*n* = 13). Animals were randomly assigned to the experimental groups.

### 4.3. Cyclosporine A

A stock solution of 100 mM cyclosporine A (Sigma-Aldrich Merck Life Science S.L.U., Madrid, Spain) in ethanol was prepared and then diluted in the perfusion solution to a final concentration of 50 µM, similar to previous studies [52]. This single concentration was used for both the electrophysiological recording experiments and the eye topical application (10 µL drops) performed in the blinking and tearing measurements.

### 4.4. Recording of the Electrical Activity of Corneal Cold Thermosensitive Nerve Terminals

Four weeks after surgical removal of the main lacrimal gland, the animals were sacrificed using an overdose of sodium pentobarbitone (120 mg/kg, i.p.). The eyeballs were removed, and the corneas were cut circularly at the limbus with iris scissors, placed in a perfusion chamber, and secured with insect pins to the silicone bottom of the chamber (Sylgard 184, Dow Corning, Midland, MI, USA). Corneas were continuously superfused at a flow of 2.5 mL/min with physiological solution (composition in mM: 133.4 NaCl; 4.7 KCl; 2 CaCl_2_; 1.2 MgCl_2_; 16.3 NaHCO_3_; 1.3 NaH_2_PO_4_ and 7.8 glucose; gassed with 95% O_2_/5% CO_2_ to pH = 7.4). The temperature of the bath solution was maintained at 34 °C (basal temperature) by means of a feedback-regulated, homemade Peltier device. Nerve terminal impulse (NTI) activity of single cold thermoreceptor terminals was recorded following the technique described by Brock et al. [58] using 50 µm-diameter glass micropipette electrodes filled with the same saline solution and applied onto the surface of the cornea under slight suction. Electrical signals were recorded with respect to an Ag/AgCl pellet in the bath, amplified (1000×, NL 103 AC amplifier, Digitimer, Welwyn, UK), filtered (high pass 1 Hz, low pass 5 KHz; NL 125/NL 126 filter, Digitimer), and stored in a computer using an interface (CED micro1401 MK II, Cambridge Electronic Design, Cambridge, UK) and dedicated software (Spike2 v.8.02, also from Cambridge Electronic Design). Only NTIs originating from single cold-sensitive terminals were selected for further study. They were identified by their relatively high level of regular spontaneous discharge, occasionally in bursts, that increased distinctly with cooling and silenced with warming of the superfusion solution [54,59,60,61]. Spontaneous NTI activity at the basal mean temperature was recorded for at least 2 min before applying a cooling ramp from 34 °C to 15 °C for a 30 s duration, obtained by decreasing the temperature of the perfusion solution at a cooling rate of −0.5 °C/s). The effect of CsA on the spontaneous and stimulus-evoked activity of cold receptors was tested by adding the test drug to the perfusion solution at 50 µM concentration during 10–15 min. Response to the cooling ramp was tested before and during exposure to the drug. In a set of experiments, the spontaneous and cold-evoked activity was also explored 15–30 min after washing the drug with a physiological solution. Different parameters were analyzed to describe the NTI activity of single cold thermoreceptor terminals (see Section 4.5), and then compared data were obtained in corneas from tear-deficient and non-operated control animals.

### 4.5. Electrophysiological Recording Analysis

**Figure 3:**
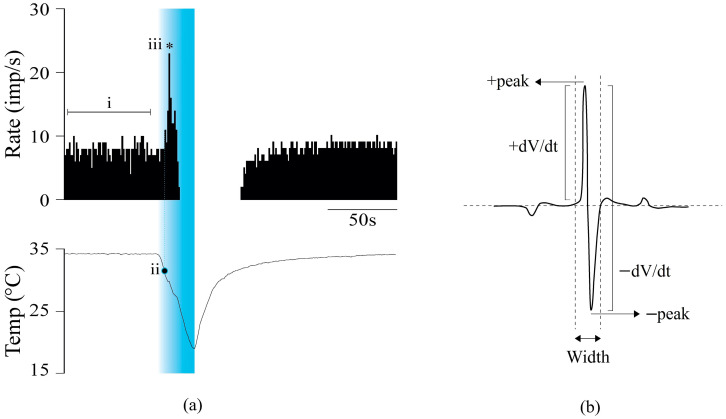
Recording experimental protocol and parameters measured. (**a**) Example of the change in NTI activity evoked by cold in a corneal nerve terminal. Upper trace: histogram of the firing frequency in imp/s. Lower trace: recording of the temperature of the perfusion solution in °C; (i) Spontaneous activity at basal temperature; (ii) Cooling threshold; (iii) Peak response to cold, that is, the maximum frequency per second during the cooling ramp reached at the time indicated by the asterisk. The blue band has been added to help visualizing the terminal discharge rate during the cooling ramp. (**b**) NTI shape parameters measured during the spontaneous activity (see Section 4.5).

NTIs were discriminated during the acquisition process using a threshold criterion and filtered manually afterward to remove electrical artifacts. The following parameters were calculated to define the spontaneous and cold-evoked firing: (i) *Spontaneous activity at basal temperature*, the average number of NTIs recorded per second (impulses/s) during 1 min at basal temperature of 34 °C; (ii) *Cooling threshold*, temperature change (in °C) during the cooling ramp at which a 25% increase in the mean NTI frequency at basal temperature was obtained; (iii) *Peak response to cold*, maximal impulse/s value of NTI frequency during the cooling ramp (Figure 3a).

NTI recorded in the present experiments were diphasic (positive–negative), often with a prominent positive component. It is assumed that extracellular potential changes recorded as NTI are produced by net membrane current variations, with positive and negative deflections from baseline being produced by net outward and inward current, respectively; thus, the NTI is the first derivative of the membrane voltage change [28]. The amplitude of the NTI varied along the recording of a single terminal, decreasing when cooling and increasing when heating [58]. So, the analysis of the shape of the NTI was based on a previous study, where the shape parameters were studied during spontaneous firing at a basal temperature [58]. To determine the effects of CsA on NTI shape, the following parameters were analyzed: (i) *Positive peak amplitude* (+peak, in mV); (ii) *Negative peak amplitude* (–peak, in mV); (iii), *Maximum rate of voltage change during the initial upstroke of the NTI* (+dV/dt max; in µV/ms); (iv) *Maximum rate of voltage change during the downstroke of the NTI* (−dV/dt max; in µV/ms); (v) *Duration of the NTI* (total width of the NTI, in ms) (Figure 3b). Because the amplitude of the NTI also varied a lot between experiments, the maximum rate of voltage change during the initial upstroke and the downstroke was normalized with respect to the NTI positive peak amplitude in order to compare different units.

### 4.6. Blinking Frequency and Tearing Rate in Awake Guinea Pigs

The blinking rate was measured as a quantitative assessment of pain (nocifensive response). This parameter can be easily determined in the awake guinea pig and reflect the eye irritation-related behavior of the animal [60,61]. Blinking was assessed in real time, watching the animal freely moving in an open-ceiling 90 × 60 × 60 cm cage for 5 min. Immediately afterward, the tearing rate was measured in both eyes using commercial phenol red threads (Zone-Quick; Menicon, Nagoya, Japan) placed in the nasal side of the lower lid for 30 s. The measurements were taken before, immediately after topical treatment with CsA, and 10 min afterward.

### 4.7. Statistical Analysis

Statistical comparisons were made using Statistica 12.0 (StatSoft Inc., Tulsa, OK, USA). Previous analysis of sample size and test power was required by the CEIR of the University of Miguel Hernández to approve the use of animals in research. Data were expressed as mean ± standard error of the mean (SEM), n being the number of studied nerve terminals or animals. Data were compared using parametric or their equivalent non-parametric tests, as indicated. The significance level was set at *p* < 0.05 in all statistical analyses.

## Figures and Tables

**Table 1:** Spontaneous activity at 34 °C and characteristics of the cold-evoked response of cold nerve terminals recorded in control and tear-deficient corneas before and during perfusion with cyclosporine A (CsA).

	Control Corneas (*n* = 7)		Tear-Deficient Corneas (*n* = 7)	
	Before	During CsA	Before	During CsA
Spontaneous activity at 34 °C (imps/s)	6.4 ± 0.9	6.4 ± 0.8	10.2 ± 0.3	11.4 ± 1.1
Cooling threshold (°C)	32.3 ± 0.4	31.7 ± 0.5	32.4 ± 0.2	32.4 ± 0.4
Peak response to cold (imp/s)	28.4 ± 5.4	21.6 ± 3.5	32.7 ± 4.5	23 ± 4.2 *

*n* = number of nerve terminals: *: *p* < 0.05, paired *t*-test compared to Before.

**Table 2:** Effects of CsA on the shape of nerve terminal impulses recorded in control and tear-deficient corneas.

Parameters of NTI Shape	Control Corneas (*n* = 7)		Tear-Deficient Corneas (*n* = 3)	
	Before	During CsA	Before	During CsA
+peak (µV)	20.97 ± 2.54	25.62 ± 4.28	16.72 ± 1.47	15.49 ± 0.59
−peak (µV)	21.57 ± 2.63	26.10 ± 3.86	17.57 ± 1.86	16.56 ± 0.37
Duration (ms)	2.75 ± 0.14	2.85 ± 0.20	2.13 ± 0.12 *	2.27 ± 0.19
+dV/dt max normalized (µV/ms)	1853 ± 227	1766 ± 257	2033 ± 251	1788 ± 1.7
−dV/dt max normalized (µV/ms)	2207 ± 202	2222 ± 191	2472 ± 233	2512 ± 190

*n* = number of terminals. * *p* < 0.05, *t*-test compared with control.

**Table 3:** Blinking and tearing rate measured before and after topical instillation of a 50 µM CsA drop.

	Control Eyes (*n* = 24)		Tear-Deficient Eyes (*n* = 6)	
	Before	During CsA	Before	During CsA
Blinking frequency (blinks/min)	1.7 ± 0.4	21.0 ± 1.6 **	10 ± 3.7	14.7 ± 2.3
Tearing rate (mm)	12.8 ± 1.2	24.0 ± 1.6 **	4.0 ± 1.0	7.8 ± 2.3

*n* = number of terminals. ** *p* < 0.001, paired *t*-test compared to before.

## Data Availability

All relevant data are within the manuscript.
